# The association and predictive value analysis of metabolic syndrome on diastolic heart failure in patients at high risk for coronary artery disease

**DOI:** 10.1186/1758-5996-5-30

**Published:** 2013-06-24

**Authors:** Zi-Hui Tang, Fangfang Zeng, Zhongtao Li, Yibing Si, Linuo Zhou

**Affiliations:** 1 Department of Endocrinology and Metabolism, Huashan Hospital of Fudan University, NO. 12 Wulumuqi Mid Road, Building 0#, Jing’an District, Shanghai 200040, China; 2 Department of Cardiology, Huashan Hospital of Fudan University, NO. 12 Wulumuqi Mid Road, Building 0#, Jing’an District, Shanghai 200040, China

**Keywords:** Metabolic syndrome, Diastolic heart failure, High-risk patients, Association, Coronary artery disease

## Abstract

**Background:**

The purpose of the present study was to evaluate the effect and predictive value of metabolic syndrome (MetS) and its components on diastolic heart failure (DHF) in patients at high risk for coronary artery disease (CAD).

**Materials and methods:**

We enrolled 261 patients with normal left ventricular ejection fraction (≥50%) who were scheduled to undergo coronary angiography for suspected myocardial ischemia. They were categorized into three groups (non-MetS, pre-MetS and MetS) based on the number of MetS criteria. Echocardiography was used to assess left ventricular (LV) diastolic function. The association between MetS and DHF was assessed by multivariate logistic regression (MLR) analysis (non-DHF patients as reference group) after controlling for confounders. The predictive performance of the MetS severity score (MSS) was evaluated using the area under the receiver-operating characteristic curve (AUC).

**Results:**

A tendency toward increased DHF prevalence with increasing MSS was found (p < 0.001). MLR analysis showed that in patients with an MSS of 1, the odds ratio (OR) of DHF was 1.60 (95% confidence interval-CI, 1.19–2.16; p = 0.02) compared to non-DHF patients; in patients with MSS ≥4, the OR was 6.61 (95% CI, 4.90–8.90; p < 0.001) compared to non-DHF patients. MSSs strongly predicted DHF (AUC = 0.73, 95% CI, 0.66–0.78, p < 0.001). MLR with MetS components as binary variables showed that blood pressure (BP) and triglycerides (TGs) were significantly associated with DHF (P = 0.001 and 0.043, respectively).

**Conclusion:**

Our findings signify that MetS and its components of BP or TG were associated with DHF in high-risk CAD patients. DHF prevalence tends to increase with increasing MSS that has a high value in predicting DHF in high-risk CAD patients.

## Background

Metabolic syndrome (MetS) is a global health concern and refers to a constellation of the risk factors of cardiovascular disease, including obesity, abdominal fat distribution, disorders of glucose and lipid metabolism and hypertension (HT) [1]. The burden of MetS is likely to continue to rise, largely due to the decrease in physical activity and increase in obesity in our society [2]. Diastolic heart failure (DHF) refers to a decline in the performance of one or both ventricles of the heart during diastole and is characterized by elevated diastolic pressure in the left ventricle (LV), despite an essentially normal end-diastolic volume [2]. Studies have suggested that the morbidity and mortality of DHF are similar to those of systolic heart failure [3,4]. DHF is a powerful and independent predictor of death [5]. DHF can be attributed to multiple factors that are mainly linked to metabolic disturbances [4,5]. Some components of MetS– such as hypertension and fasting blood glucose are strongly associated with DHF, which leads to stiffening of the LV, resulting in diastolic dysfunction [4,5]. Moreover, hypertension, diabetes mellitus (DM) and obesity have been found to adversely affect cardiac structure and function [6,7].

Patients who are at a high risk for coronary artery disease (CAD) characteristically have HT, DM and hyperlipidemia. MetS and DHF tend to be co-prevalent in high-risk CAD patients, who account for more than half of the patients hospitalized in departments of cardiovascular disease [8]. High-risk CAD patients with DHF have been found to have high morbidity and mortality. It is important to clarify the relationship between MetS and DHF in high-risk CAD patients, as this information can be of benefit to clinicians in the prediction, prevention and treatment of DHF. However, the extent to which the clustering of MetS components predicts DHF in high-risk CAD patients is unknown. In addition, the effects of MetS and its components on DHF have not been well characterized in this subgroup of patients. The purpose of the present study was to evaluate the effect and predictive value of MetS and its components on DHF in high-risk CAD patients.

## Materials and methods

### Study population

Two hundred and sixty-one consecutive symptomatic or asymptomatic Chinese patients who had normal LV ejection fraction (LVEF; ≥50%) and were scheduled to undergo coronary angiography for suspected myocardial ischemia were recruited between February 2009 and March 2011 at the Huashan Hospital of Fudan University, China. Patients were excluded from the study to eliminate potential confounding factors that may have influenced heart function. The exclusion criteria were as follows: (1) history or findings of systolic heart failure (LVEF ≤ 45%), significant valvular heart disease (i.e., more than a mild valvular insufficiency or stenosis), hyperthyroidism or hypothyroidism and dilated or hypertrophic cardiomyopathy, (2) pregnancy or lactation and/or (3) a major systemic illness, such as systemic lupus erythematosus. Written consent was obtained from all patients before the study, which was performed in accordance with the ethical standards laid down in Declaration of Helsinki and approved by the Medicine Ethical Committee of Fudan University.

MetS was diagnosed according to the updated National Cholesterol Education Program/Adult Treatment Panel III criteria (WHO Western Pacific Region obesity criteria) in individuals meeting three or more of the following [9]: (1) central obesity, defined using ethnicity-specific values as a waist circumference (WC) of ≥90 cm in men or ≥80 cm in women; (2) raised triglyceride (TG) levels, >150 mg/dl (1.7 mmol/l) or specific treatment for this lipid abnormality; (3) reduced high-density lipoprotein (HDL) cholesterol, <40 mg/dl (1.03 mmol/l) in men and <50 mg/dl (1.29 mmol/l) in women or specific treatment for this lipid abnormality; (4) raised blood pressure (BP), systolic BP >130 mm Hg or diastolic BP >85 mm Hg or treatment for previously diagnosed HT; and (5) raised fasting plasma glucose (FPG) level, >100 mg/dl (>5.6 mmol/l) or previously diagnosed type 2 DM. Patients with raised FPG levels were strongly recommended to undergo an oral glucose-tolerance test. In addition, if the body mass index (BMI) was >30 kg/m^2^, central obesity was assumed, and WC did not need to be measured. For analysis, the study subjects were grouped according to the number of MetS criteria they satisfied: non-MetS (0 criteria), pre-MetS (1–2 criteria) and MetS (≥3 criteria). MetS severity was scored on a scale of 0 to 4 according to the number of MetS components present. Three subjects met all five MetS criteria, and their MetS severity score (MSS) was set to 4.

The subjects were interviewed for the documentation of medical histories, medications, history of smoking habits, laboratory assessment of cardiovascular disease risk factors and standardized echocardiographic examination. BMI was calculated as the weight in kilograms divided by the square of height in meters. SBP and DBP values were the means of two physician-obtained measurements on the left arm of the seated participant. HT was diagnosed if the BP was ≥140/90 mm Hg and/or the patient was undergoing antihypertensive therapy. DM was diagnosed on the basis of the oral glucose tolerance test and either a glycosylated hemoglobin (HbAlc) level of ≥6.5% or the use of insulin or hypoglycemic medications. CAD was diagnosed if any one of the following were present: (1) history and/or treatment for angina and/or myocardial infarction; (2) history of coronary artery revascularization procedures and/or coronary angiography with ≥50% stenosis in one or more of the major coronary arteries; and (3) regional wall-motion abnormalities on rest echocardiography.

### Laboratory assays

Peripheral venous blood samples were collected in tubes in the fasting state in all subjects. The blood was centrifuged at 3000 rpm for 10 min for plasma separation and immediately used to measure biomarkers. FPG was measured using the glucose oxidase procedure; HbA1c was measured using ion-exchange high-performance liquid chromatography (HPLC; Bio-Rad, Hercules, CA, USA). Serum total cholesterol (TC), HDL cholesterol, TGs, creatinine (Cr) and uric acid (UA) levels were measured using an enzymatic method with a chemical analyzer (Hitachi 7600–020, Tokyo, Japan). Low-density lipoprotein (LDL) cholesterol levels were calculated using the Friedewald formula, and the creatinine clearance rate (Ccr) was calculated using the Cockcroft-Gault formula. The day-to-day and inter-assay coefficients of variation at the central laboratory in our hospital for all analyses were between 1% and 3%.

### Echocardiographic measurement

Echocardiography examinations were performed with a Vingmed System 5 Doppler echocardiographic unit (GE Vingmed Ultrasound, Horten, Norway). Conventional echocardiography measurements were performed according to American Society of Echocardiography guidelines. LV mass (LVM) was calculated using the Devereux formula. The LVM was corrected for body surface area (BSA) to obtain the LVM index (LVMI). The left atrial diameter (LAD) and aortic root dimension (AOD) were also measured. LV systolic function was assessed using the LVEF. Diastolic function was assessed by determining the E-to-A ratio (E/A) and deceleration time (DT), where E and A represent the early and late ventricular filling velocities, respectively.

We used the definition of DHF recommended in the European Society of Cardiology guidelines in 2008 [10]. The diagnosis of DHF required the following three conditions: (1) presence of signs and/or symptoms of chronic heart failure meanly involved in shortness of breath, rapid heartbeat, wheezing or abnormal heart sounds and swollen legs; (2) presence of normal or only mildly abnormal LV systolic function (LVEF, 45%–50%); and (3) evidence of diastolic dysfunction (abnormal LV relaxation or diastolic stiffness). Diastolic function of the LV was evaluated on the basis of the ventricular filling pattern in patients with heart failure. Normal LV diastolic function was defined as an E/A ratio >1 and 160 ms < DT < 240 ms. LV diastolic dysfunction was defined as (1) E/A ratio <1 and DT ≥260 ms or (2) E/A ratio >2 and DT <150 ms.

### Statistical analysis

The Kolmogorov-Smirnov test was used to determine whether continuous variables followed a normal distribution. Variables that were not normally distributed were log-transformed to approximate normal distribution for analysis. The results are expressed as the mean ± SD or median, unless otherwise stated. The characteristics of the subjects according to MetS groups were assessed using one-way analysis of variance (ANOVA) for continuous variables and the χ^2^ test for categorical variables. Univariate linear regression was performed to determine the variables associated with DHF and to estimate confounding factors possibly disturbing the relationship between MetS and DHF. Multivariate logistic linear regression (MLR) was carried out to determine the independent contributions of variables to DHF (non-DHF patients as reference group). Potential confounding variables, including age, gender, smoking, LAD, LVMI, UA, Ccr and Cr, were controlled in the regression model. Variables were entered into the backward stepwise regression models if a p value of <0.10 was obtained. The models were re-analyzed after substituting the continuous variables related to all MetS components with their dichotomous counterparts in the models. The predictive performance of the MSS was evaluated using the area under the curve (AUC) in a receiver operating characteristics (ROC) curve. Odds ratios (ORs) with 95% confidence intervals (CIs) were calculated for the relative risk of MetS with DHF. The results were analyzed using the Statistical Package for Social Sciences for Windows, version 16.0 (SPSS, Chicago, IL, USA). The tests were two-sided, and a p value of <0.05 was considered significant.

## Results

The baseline clinical characteristics of the 261 subjects were grouped according to the type of MetS (Table 1). The total sample included 153 men and 108 women (mean age, 59.56 ± 14.53 years), and 24.53% of these patients were found to have MetS. Gender, height and TC levels were similar among the three MetS groups (p > 0.05), while the other demographic parameters and biochemical variables were significantly different (p < 0.05). LVEF did not significantly differ among the three groups, but LAD, DT and LVMI did (p < 0.05). The prevalence of CAD, HT and DM were 30.3%, 59.4% and 39.1% in the patients, respectively. These three conditions were more prevalent in the MetS group than the other two groups (p < 0.05). Smoking habits were similar among the three groups, while the use of oral medications was significant different (p < 0.05).

**Table 1:** Characteristics of the study population

**Variable**	**Total (n = 261)**	**Non-MetS (n = 48)**	**Pre-MetS (n = 149)**	**MetS (n = 64)**	***p *value**
Age	59.56 ± 14.53	51.27 ± 14.45	59.68 ± 14.4	65.48 ± 11.85	<0.001
Gender (male, %)	153(58.6%)	25(52.1%)	89(59.7%)	39(60.9%)	0.588
Height	165.83 ± 8.11	165.1 ± 8.45	165.95 ± 7.77	166.08 ± 8.71	0.789
Weight	65.11 ± 11.74	58.55 ± 8.49	63.63 ± 10.87	73.47 ± 11.34	<0.001
BMI	23.56 ± 3.54	21.38 ± 2.08	22.97 ± 3.29	26.55 ± 3.11	<0.001
WC	82.92 ± 7.82	77.97 ± 6.213	82 ± 7	87.04 ± 7.19	<0.001
SBP	128 ± 18	116 ± 11	128 ± 18	136 ± 18	<0.001
DBP	76 ± 11	72 ± 7	76 ± 11	81 ± 13	<0.001
HR	71 ± 11	66 ± 11	72 ± 11	73 ± 12	0.007
Laboratory assay					
FPG	6.02 ± 2.52	4.83 ± 0.26	6.11 ± 2.88	6.73 ± 2.25	<0.001
PBG	8.48 ± 4.08	6.24 ± 0.83	8.4 ± 4.25	9.66 ± 4.32	0.004
HbAlc	6.74 ± 2.13	5.23 ± 0.42	6.91 ± 2.36	7.16 ± 1.97	0.003
TC	4.5 ± 1.12	4.21 ± 0.81	4.59 ± 1.13	4.49 ± 1.26	0.142
TG	1.61 ± 1.2	0.98 ± 0.33	1.49 ± 0.95	2.34 ± 1.68	<0.001
HDL	1.14 ± 0.3	1.3 ± 0.28	1.16 ± 0.3	0.99 ± 0.23	<0.001
LDL	2.56 ± 0.89	2.31 ± 0.67	2.67 ± 0.92	2.49 ± 0.91	0.048
Cr	76.11 ± 25.93	68.04 ± 14.06	74.84 ± 22.45	84.78 ± 35.96	0.002
Ccr	85.58 ± 30.31	91.04 ± 24	85.46 ± 31.27	81.95 ± 31.97	0.300
UA	0.34 ± 0.1	0.3 ± 0.08	0.34 ± 0.1	0.38 ± 0.12	0.001
Echocardiographic measurement					
LVEF (%)	65 ± 6	65 ± 4	66 ± 4	65 ± 6	0.426
DT	217.29 ± 56.37	196.67 ± 43.63	214.3 ± 59.54	239.7 ± 50.12	<0.001
LAD	36.17 ± 4.81	33.67 ± 4.07	35.7 ± 4.45	39.14 ± 4.75	<0.001
LVMI	112.77 ± 35.91	98.51 ± 28.23	108.46 ± 35.72	133.71 ± 33	<0.001
DHF (yes, %)	137(52.5%)	10(20.80%)	78(52.3%)	49(76.6%)	<0.001
Past medical history (yes, %)					
CAD	79(30.3%)	6(12.5%)	41(27.5%)	32(50%)	<0.001
HT	155(59.4%)	0(0.0%)	95(63.8%)	60(93.8%)	<0.001
DM	102(39.1%)	0(0.0%)	58(38.9%)	44(68.8%)	<0.001
Smoking	75(28.7%)	14(29.2%)	49(32.9%)	12(18.8%)	0.106
Medical therapy (yes, %)					
Anti-hypertension	140(53.6%)	0(0.0%)	85(57%)	55(85.9%)	<0.001
Hypoglycaemic	78(29.9%)	0(0.0%)	45(30.2%)	33(51.6%)	<0.001
Anti-lipids	69(26.4%)	5(10.41%)	41(27.5%)	23(35.9%)	0.009

Note: *BMI* Body mass index, *SBP* systolic blood pressure, *DBP* diastolic blood pressure, *MetS* metabolic syndrome, *HT* Hypertension, *DM* Diabetes, *FPG* fasting plasma glucose, *PBG* plasma blood glucose, *HbA1c* glycated hemoglobin, *TC* serum total cholesterol, *HDL* high-density lipoprotein cholesterol, *TG* triglyceride, *UA* uric acid, *LDL* low density lipoprotein cholesterol, *Ccr* creatinine clearance rate, *Cr* creatinine, *LVMI* left ventricular mass index, *LAD* left atrial diameter, *DT* deceleration time, *LVEF* left ventricular ejection fraction.

### Prevalence of DHF in high-risk CAD patients

**Figure 1:**
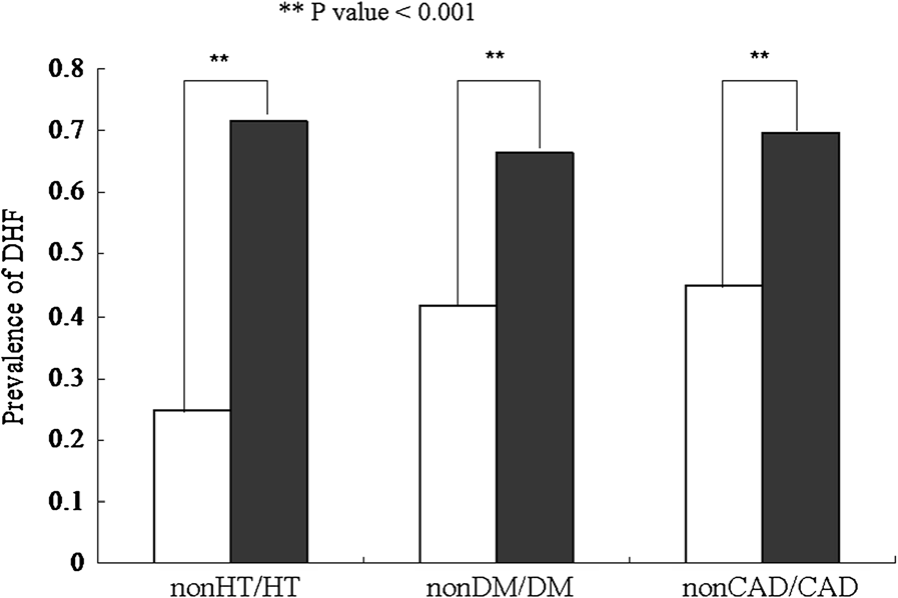
Prevalence of diastolic heart failure (DHF) in patients with hypertension (HT), diabetes mellitus (DM) and coronary artery disease (CAD).

**Figure 2:**
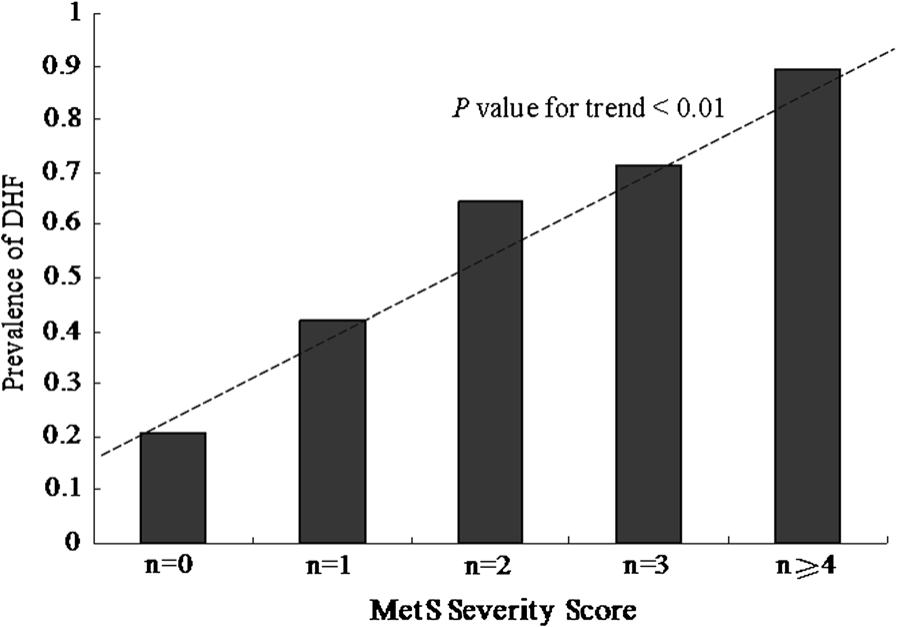
Prevalence of diastolic heart failure (DHF) according to metabolic syndrome (MetS) severity score.

The prevalence of DHF was 52.50% in the total sample and 20.80%, 52.3% and 76.6% in the non-MetS, pre-MetS and MetS groups, respectively. DHF prevalence was 71.61%, 66.37% and 69.62% in patients with HT, DM and CAD, respectively (Figure 1). DHF prevalence was significantly lower in patients without HT (24.76%) than in patients with HT (p < 0.001). Similar DHF prevalence was found in patients with DM and without DM (41.89%) and those with and without CAD (45.14%; p < 0.001 for both). In addition, DHF prevalence significantly differed with the MSS (MSS 0, 20.83%; 1, 41.77%; 2, 64.28%; 3, 71.11%; and 4, 89.47%; p < 0.01; Figure 2). As the MSS increased, the prevalence of DHF also increased (p for trend < 0.01; Figure 2).

### MetS severity and DHF

**Figure 3:**
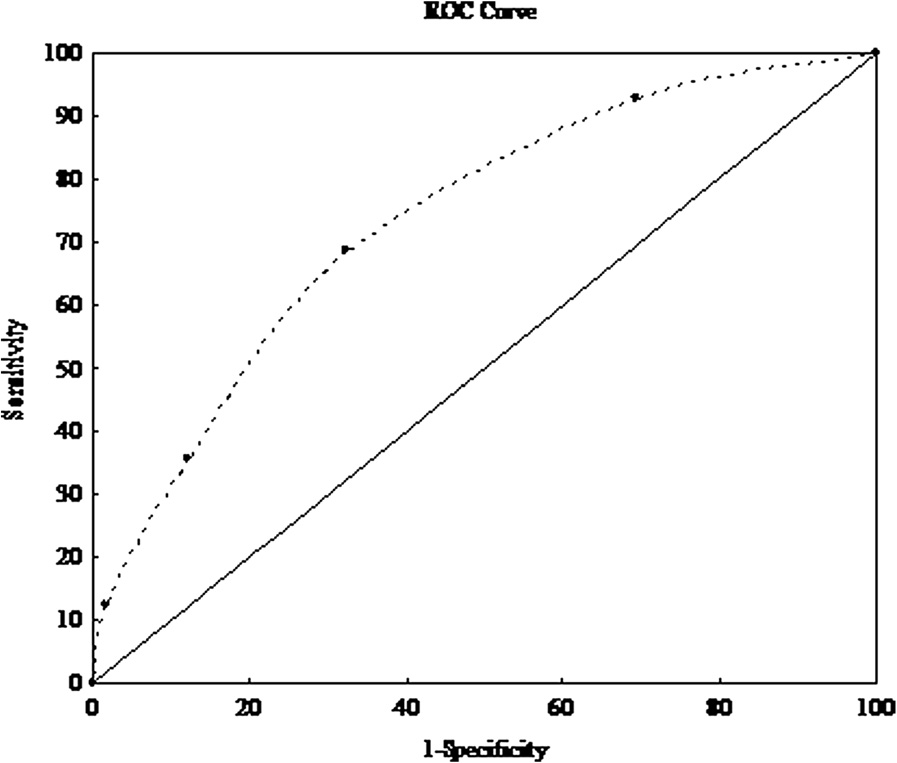
**Receiver operative characteristic (ROC) curve showing the performance of metabolic syndrome (MetS) severity score in predicting the prevalence of diastolic heart failure (DHF).** Area under the curve (AUC) = 0.726, (95% confidence interval, 0.665–0.787), p < 0.001.

To estimate the association of MetS severity and other factors with DHF, univariate logistic regression models were developed to include gender, age, height, weight, BMI, WC, SBP, DBP, FPG, TG, HDL, other lipid profiles, Cr, Ccr, UA, echocardiographic parameters of LV, past medical history and medical therapy (Table 2). The univariate analyses indicated that age, height, Cr, Ccr, UA, LVMI, CAD, DM, HT, MetS and all its components, except HDL, were significantly associated with DHF (p < 0.05 for all). MLR analysis was carried out to determine the extent to which DHF was associated with MetS severity. MetS remained significantly associated with DHF after adjustments for age, height, Cr, Ccr, UA, LVMI, HT, DM and CAD (p = 0.023, data not shown). In patients with an MSS of 1, the OR of DHF was 1.60 (95% CI, 1.19–2.16; p = 0.02; Table 3) compared to non-DHF patients, while in patients with an MSS ≥ 4, the OR was 6.61 (95% CI, 4.90–8.90; p < 0.001) compared to non-DHF patients. To evaluate the predictive performance of MSSs for DHF, the AUC in an ROC curve was calculated. The AUC was 0.726 (95% CI, 0.665–0.787; p < 0.001), indicating that the MSS strongly predicted DHF (Figure 3) .

**Table 2:** Univariate regression analysis for diastolic heart failure

**Variables**	***β***	**S.E.**	***P *value**	***OR***	**95.0% C.I.**
BMI	0.60	0.27	0.03	1.82	1.06-3.14
SBP	0.05	0.01	<0.001	1.06	1.04-1.08
DBP	0.03	0.01	0.003	1.03	1.01-1.06
FPG	0.83	0.50	0.010	2.29	1.85-6.17
TG	0.69	0.28	0.014	2.00	1.15-3.47
HDL	−0.47	0.43	0.269	0.62	0.27-1.45
MetS	0.78	0.13	<0.001	2.20	1.70-2.84
Age	0.86	0.11	<0.001	2.36	1.87-2.99
Ccr	−0.02	0.01	<0.001	0.97	0.97-0.98
Cr	0.02	0.01	<0.001	1.02	1.01-1.04
UA	1.38	0.37	<0.001	4.00	1.94-8.26
LVMI	0.02	0.01	<0.001	1.02	1.02-1.03
HT	2.03	0.28	<0.001	7.66	4.36-13.48
DM	1.01	0.26	<0.001	2.73	1.65-4.55
CAD	1.02	0.28	<0.001	2.78	1.58-4.90

Note: *MI* Body mass index, *SBP* systolic blood pressure, *DBP* diastolic blood pressure, *MetS* metabolic syndrome, *HT* Hypertension, *DM* Diabetes, *FPG* fasting plasma glucose, *PBG* plasma blood glucose, *HbA1c* glycated hemoglobin, *TC* serum total cholesterol, *HDL* high-density lipoprotein cholesterol, *TG* triglyceride, *UA* uric acid, *LDL* low density lipoprotein cholesterol, *Ccr* creatinine clearance rate, *Cr* creatinine, *LVMI* left ventricular mass index, *LAD* left atrial diameter, *DT* deceleration time, *LVEF* left ventricular ejection fraction.

**Table 3:** Multiple logistic regression analysis for diastolic heart failure, including metabolic syndrome (MetS)

**MetS severity score**	**Number**	***β***	***P *value**	***OR***	**95% CI**
0 (reference)	48	0.0	---	1.00	---
1	79	0.47	0.020	1.60	1.19-2.16
2	70	0.94	<0.001	2.57	1.91-3.46
3	45	1.42	<0.001	4.12	3.06-5.55
≥ 4	19	1.88	<0.001	6.61	4.90-8.90

Note: Adjusted for age, gender, serum creatinine, creatinine clearance rate, serum uric acid, left ventricular mass index and coronary artery disease.

### MetS components and DHF

MLR analysis with the whole covariate model and backward stepwise regression model with adjustments for confounders were developed to include MetS components as continuous variables. The enter regression model after adjustments for age, Cr, Ccr, UA, LVMI and CAD indicated that DBP was significantly associated with DHF (p = 0.022; data not shown). The backward stepwise regression model showed that age, UA, LVMI and DBP were independently associated with DHF (p = 0.001, 0.041, 0.044 and 0.001, respectively; data not shown). To reduce the effect of medical therapy, MLR models with adjustments for confounders were developed to include MetS components as binary variables. The results showed that BP and TG were significantly associated with DHF (p = 0.001 and 0.043, respectively in the enter regression model; p = 0.001 and 0.038, respectively in the backward stepwise regression model; Table 4).

**Table 4:** Multiple logistic regression analysis for diastolic heart failure (DHF) and components of metabolic syndrome (MetS) as binary variables

**Model**	**Variable**	***β***	**S.E.**	***P *value**	***OR***	**95% CI**
Model 1	BMI	0.12	0.407	0.773	1.125	0.51-2.5
	BP	1.82	0.428	<0.001	6.197	2.68-14.35
	FBG	0.47	0.364	0.201	1.592	0.78-3.25
	TG	0.80	0.416	0.043	2.236	1.01-5.05
	HDL	−0.36	0.376	0.339	0.698	0.33-1.46
Model 2	Constant	−3.41	0.514	<0.001		
	Age	0.69	0.146	<0.001	2.004	1.51-2.67
	UA	0.93	0.488	0.045	2.546	1.08-6.62
	BP	1.93	0.413	<0.001	6.893	3.07-15.49
	TG	0.81	0.388	0.038	2.236	1.05-4.78

Note: Model 1 adjusted for age, serum creatinine, creatinine clearance rate, serum uric acid, left ventricular mass index and coronary artery disease; Model 2 was the best model. The following variables were entered in step 1: age, serum creatinine, creatinine clearance rate, serum uric acid, left ventricular mass index and coronary artery disease, body mass index, blood pressure, fasting blood glucose, triglycerides and high-density lipoprotein.

*SE* standard error, *OR* odds ratio, CI confidence interval.

## Discussion

We first conducted a case–control study to evaluate the effect of metabolic factors on DHF in Chinese high-risk CAD patients. CAD, HT and DM were more prevalent in patients with MetS. Most of the demographic factors, biochemical characteristics and echocardiographic measurements significantly differed among the three MetS groups. Doppler echocardiography has become a well accepted, reliable, noninvasive tool to measure the LV diastolic function. In the present study, Doppler echocardiography was used to measure LV diastolic function in order to diagnose DHF.

The main finding of the present study was that MetS strongly and independently predicted DHF in high-risk CAD patients. The prevalence of DHF increased with the severity of MetS. HT, insulin resistance and obesity have been associated with LV diastolic dysfunction or DHF in different populations [11,12]. In addition, MetS has been independently correlated with DHF in different subgroups such diabetic, non-diabetic or hypertensive patients [13-15]. In the present study, the association between MetS and DHF was observed in both the univariate and multivariate models after adjustment for potential confounders in high-risk CAD patients. Specifically, we found a good association between the MSSs and DHF. To our knowledge, this is the first study to have reported such an association in a population of high-risk CAD patients. In the multivariate analysis, MetS was independently associated with DHF, even after adjustment for potential confounders such as parameters of renal function, LVMI and CAD. This finding is of special importance if the direct relationship between MetS and DHF is considered. The clustering of cardiovascular risk factors in MetS indicates that the multiple complex metabolic reactions involved in glycotoxicity, lipotoxicity, altered insulin signaling, increased cytokine activity and interstitial deposition of triacylglycerol may directly or indirectly impact myocardial function [4,16-20]. Additionally, these metabolic risk factors lead to reduced energy availability, and have an additive, adverse effect on endothelial function [21].

In the present study, the AUC was calculated to show that the MSS strongly predicts DHF. In patients with MSSs of up to 4, the prevalence of DHF was nearly 90%. This finding indicates that the severity of MetS is linked to the progression of DHF. This is one of interesting findings of the present study, which not only supports further studies on the mechanism of DHF but also provides evidence for clinicians to predict DHF in hospitalized patients. However, in the present study, we scored the MetS severity by simply using the number of MetS criteria. We did not consider the weights of the MetS components. For instance, the BP component of MetS makes a greater contribution to DHF. A large-scale, case–control study or cohort study with a better method of scoring MetS severity will be conducted to develop a highly sensitive and specific model that uses MetS information to predict DHF. Such a model would facilitate the prevention and treatment of DHF in clinical practice.

Another interesting finding of the present study was that BP and TG were the only MetS components that contributed to DHF. This finding is inconsistent with those of some earlier studies, which had revealed that BMI, SBP, DBP and lipid profiles were significantly associated with diastolic parameters and the structure and functions of the LV [4,16-18,20]. In the present study, BMI, FPG and HDL were not significantly associated with DHF. This difference is partly because the contributions of individual MetS components could not be detected in the present study, which had a moderate sample size. Another possible cause is that the present study population differed from those in previous studies; we performed association analysis for MetS and DHF in high-risk CAD patients with HT, DM, CAD and hyperlipidemia, which were potential confounders of DHF. In addition, MetS components as continuous variables may not reflect the true values in patients undergoing medical therapy. For example, the FPG values measured in the present study were less than the true values in DM patients using hypoglycemic drugs. However, we focused on metabolic factors associated with DHF in a specific subgroup, high-risk CAD patients. Our findings will provide evidence for clinicians to better understand and treat patients in this specific subgroup. Nevertheless, further studies should explore the effects of MetS on DHF in an exclusive subgroup such as patients with CAD or hyperlipidemia. DBP as a continuous variable was found to be associated with DHF in both the univariate and multivariate models. The results were confirmed in the MLR analysis with BP as a binary variable. DBP has been found to be an important predictor of LV diastolic dysfunction or DHF [20,22]. DBP can directly influence diastolic function and remodel the LV structure, leading to DHF [23]. In the present study, TG as a binary variable was associated with DHF in both the univariate and multivariate analyses. Other studies have reported similar results [11,24]. No consistent results have been found in MLR analyses with TG as a continuous variable. This is partly because high-risk CAD patients were regularly treated with anti-lipids drugs such as statins to prevent events of cardiovascular disease; this may have influenced the true value of TG, making it difficult to determine the effect of TG on DHF. The exact mechanism underlying the association between TG and DHF has not been fully elucidated. In the present study, we did not determine the mechanism via which TG modifies metabolic factors and induces DHF.

Several limitations of the study deserve comment. First, the design of the present study was hospital-based, which is susceptible to selection bias. Second, the sample size was moderate, limiting its ability to detect significant results. Third, the multiple regression models indicated only a moderate influence of MetS on DHF. Other environmental and genetic factors may contribute to the unexplained variation in DHF prevalence. Fourth, the association between insulin resistance and DHF was not analyzed in the present study. This is because data on fasting blood insulin levels were missing. Furthermore, most of participants were enrolled with the first diagnosis of diastolic heart failure or not. So we did not collect information of the history of diastolic heart failure. Finally, it is important to mention that our study was performed on Chinese individuals, and our findings may not be relevant to people of other ethnicities.

## Conclusion

In conclusion, individuals with MetS frequently have a higher prevalence of DHF. Our findings signify that MetS is an independent predictor of DHF, and BP and TG, as components of MetS, are independently associated with DHF. There is a tendency toward increased prevalence of DHF with increasing MSSs. This supports the hypothesis that MetS is involved in the regulation of DHF progression. The present observations provide evidence that improved metabolic control may coordinately and perhaps even synergistically inhibit the progression of DHF and also provide novel insights into biological functions, in the future.

## Abbreviations

ACEI: Angiotensin-converting enzyme inhibitor; Alb/Cr: Albumin/urinary creatinine ratio; AOD: Aortic root dimension; BMI: Body mass index; BSA: Body surface area; Ccr: Creatinine clearance rate; CI: Confidence intervals; Cr: Creatinine; DBP: Diastolic blood pressure; DHF: Diastolic heart failure; DM: Diabetes; DT: Deceleration time; E/A: E-to-A ratio; FPG: Fasting plasma glucose; GLM: General linear model; HbAlc: Glycosylated hemoglobin; HDL: High-density lipoprotein cholesterol; HOMA-IR: Homeostasis model assessment insulin resistance estimate; HT: Hypertension; IDF: International diabetes federation; LAD: Left atrial diameter; LDL: Low-density lipoprotein cholesterol; LV: Left ventricle; LVEF: Left ventricular ejection fraction; LVM: Left ventricular mass; LVMI: Left ventricular mass index; MetS: Metabolic syndrome; MLR: Multivariable logistic linear regression; OGTT: Oral glucose tolerance test; OR: Odds ratios; PBG: Postprandial blood glucose; HT: Hypertension; SBP: Systolic blood pressure; TC: Serum total cholesterol; TG: Triglyceride; WC: Waist circumference; UA: Uric acid.

## Competing interest

The authors declare that they have no conflicts of interest.

## Authors’ contribution

LZ and YS conceived of the study, and participated in its design and coordination and helped to draft the manuscript. Z-HT participated in the design of the study and performed the statistical analysis, carried out the molecular genetic studies, participated in the sequence alignment and drafted the manuscript. FZ and ZL carried out the molecular genetic studies, participated in the sequence alignment and drafted the manuscript. All authors read and approved the final manuscript.
